# Implementing psychosocial support models in contexts of extreme adversity: lessons from a process evaluation in Colombia

**DOI:** 10.3389/fpsyg.2023.1134094

**Published:** 2023-05-22

**Authors:** Arturo Harker Roa, Natalia Córdoba Flechas, Andrés Moya, María Pineros-Leano

**Affiliations:** ^1^School of Government and IMAGINA Research Center, Universidad de los Andes, Bogota, Colombia; ^2^IMAGINA Research Center, Universidad de los Andes, Bogota, Colombia; ^3^Department of Economics, Universidad de los Andes, Bogota, Colombia; ^4^School of Social Work, Boston College, Chestnut Hill, MA, United States

**Keywords:** early childhood, implementation science, process evaluation, community violence, forced displacement and migration, mental health, child development, psychosocial support

## Abstract

**Introduction:**

High quality investments during early childhood allow children to achieve their full potential by setting developmental foundations. However, challenges in the scale-up of evidence-based interventions make across-the-board implementation a non-trivial matter. Moreover, extreme contextual conditions -such as community violence, forced displacement, and poverty- impose a double threat. First, by directly affecting early childhood development (ECD), forced displacement and exposure to violence during early childhood, coupled with deficits in nurturing relationships, can trigger toxic stress, affecting children’s mental health and social and emotional learning. Second, contexts of extreme adversity exacerbate common implementation pitfalls in the scale-up of interventions. Recognizing and documenting “what it takes” to successfully implement “what works” can contribute to the expansion and effectiveness of evidence-based programs that promote ECD in these settings. *Semillas de Apego* (SA, onward), a community-based psychosocial support model for caregivers, materialized as a strategy to promote ECD in communities affected by violence and forced displacement.

**Methods:**

This article presents the results of the process evaluation of SA during the 2018–2019 implementation in Tumaco, a violence ridden municipality in the south-west border of Colombia, South America. In this phase, the program reached 714 families, 82% direct victims of violence and 57% were internally displaced. The process evaluation combined qualitative and quantitative methodological approaches to produce evidence of factors that promoted implementation quality.

**Results:**

Findings identified salient components of the program that promoted the program’s acceptability, adoption, appropriateness, fidelity and sustainability: a rigorous cultural adaptation; well-structured team selection and training methodologies; and a team support and supervision protocol to provide continuous capacity building and prevent burn-out and other occupational hazards common among professionals in mental health and psychosocial support interventions. The statistical analysis using monitoring data identified key predictors of the dosage delivered (a measure of fidelity). Evidence suggests that initial attendance to the program and observable characteristics -such as educational attainment, violence victimization and employment status-predict a successful compliance (in terms of dosage to benefit from the program).

**Discussion:**

This study provides evidence for the development of structural, organizational, and procedural processes for the adoption, appropriate adaptation, and high-fidelity delivery of psychosocial support models delivered in territories affected by extreme adversity.

## 1. Introduction

Public policy efforts focused on early childhood are one of the most cost-effective mechanisms to end transmission of poverty across generations and successfully reduce socioeconomic inequalities globally (Noble, 2021). High quality investments during this critical period -from birth to age 5- allow children to achieve their full potential by setting developmental foundations that determine a successful life trajectory in terms of educational, health and labor outcomes (Bowles et al., 2001; Cawley et al., 2001; Heckman, 2006; Borghans et al., 2008; Black et al., 2017). However, challenges in the scale-up of evidence-based interventions make across-the-board implementation a non-trivial matter (Aboud et al., 2018). Moreover, extreme contextual conditions -such as community violence, forceful displacement and poverty- impose a double threat. First, by directly affecting early childhood development (ECD), forced displacement and exposure to violence during early childhood, coupled with deficits in nurturing relationships, can trigger toxic stress, affect children’s mental health and social and emotional learning (Shonkoff and Phillips, 2000; Felitti, 2002; Walker et al., 2007; Evans and Schamberg, 2009; Blair, 2010; Molano et al., 2018). Second, contexts of extreme adversity, with low resources, exacerbate common implementation pitfalls in the scale-up of interventions (Al-Ubaydli et al., 2021). Recognizing and documenting “what it takes” to successfully implement “what works” can contribute to the expansion and effectiveness of evidence-based programs that promote ECD in these settings (Gupta et al., 2021).

It is estimated that, by 2021, a total of 36.6 million children in the world have been afflicted by forceful displacement caused by conflict, violence and other crises (UNHCR, 2022), and 449 million children –i.e., one in six children in the world– were living in a conflict zone (Strømme et al., 2022). In Colombia, more than two hundred thousand children between 0 and 5 years of age have been officially registered as victims of the internal armed conflict, and approximately three hundred thousand children have fled with their families from the Venezuelan social and economic crisis and arrived to the country (R4V, 2022; RUV, 2022). This constitutes a latent mechanism for the transmission of poverty and inequality across generations and should set as a priority the design and implementation of evidence-based programs that effectively promote ECD in conflict-affected, low-resourced settings (Ibáñez and Moya, 2010).

In 2014, *Semillas de Apego* (SA, onward), a community-based psychosocial support model for caregivers, materialized as an intervention to promote ECD in communities affected by violence and forced displacement in Colombia. SA promotes maternal mental health through the improvement of healthy and nurturing child–parent relationships, which ultimately foster ECD and overall well-being. This article presents the results of the process evaluation of SA during the 2018–2019 implementation in Tumaco, a violence ridden municipality in the south-west border of Colombia. This work adds to the scarce literature identifying implementation barriers and enablers of ECD interventions in low-resourced settings such as Brazil (Gonçalves et al., 2019; Buccini et al., 2021) and Turkey (Erdemir, 2022a; Erdemir, 2022b), and contributes evidence from a context characterized by community violence, armed conflict and forced displacement.

This study is conceptually framed by the final stage of the “translational pipeline” model, which collects research that focuses on understanding how to guarantee that an intervention with previously proven effectiveness will work at a larger scale, at similar settings (scale-up) or at somehow different settings (scale-out) (WHO and ExpandNet, 2010). This study focuses on identifying key determinants of “implementation effectiveness” (or implementation success) of an innovative psychosocial support intervention that has a proven “treatment effectiveness” (or intervention success) in a community exposed to recurring violence and forced displacement. Specifically, qualitative and quantitative methods were used in the study to answer the following questions: (1) which practices promoted *implementation success* of SA in Tumaco during 2018 and 2019?; (2) what factors should be considered essential during future scale-up phases to guarantee implementation quality?; and (3) are there any improvement opportunities in the implementation protocol to further promote implementation quality? Qualitative approaches were used to better understand specific dimensions of implementation quality – acceptability, adoption, appropriateness, feasibility, fidelity, penetration and sustainability-. Quantitative methods were used to describe mechanisms behind the level of take-up, adherence and “dosage” received by participants, as measures of fidelity in Proctor et al. (2011).

By providing evidence on factors that promoted five of the abovementioned dimensions of implementation quality, this study informs the construction of structural, organizational, and procedural processes for future adaptation and high-fidelity delivery of psychosocial support models in territories affected by community violence and forced displacement. Promoting ECD and overall wellbeing of children living in similar contexts of extreme adversity should be one of the global key priorities, where the number of forcibly displaced families has more than doubled in the last decades and now includes over 30 million.

## 2. Materials and methods

### 2.1. Semillas de Apego: Theory of change and implementation teams

**Figure 1:**
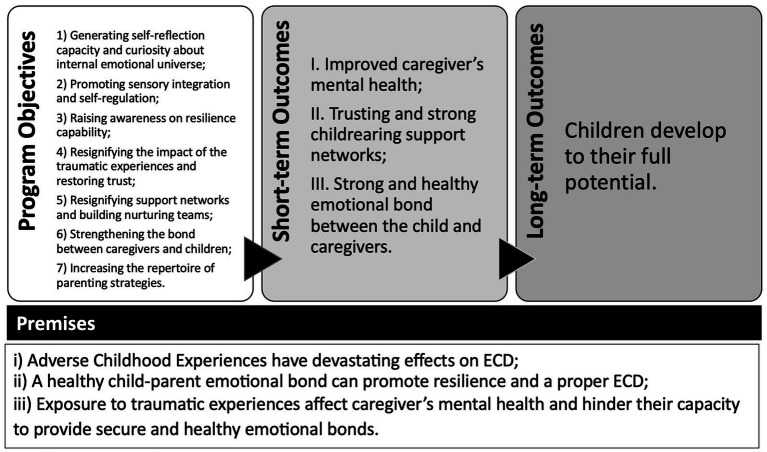
Theory of change for *Semillas de Apego*.

Semillas de Apego (SA) promotes mental health and healthy child-caregiver attachment as a pathway for a proper development among children exposed to community violence and forced displacement. By providing psychosocial support to primary caregivers’, the program aims to help children reach their full potential amid toxic stress. SA’s theory of change is characterized by three premises and seven objectives that lead to three short-term outcomes and one long-term outcome (see Figure 1). The three premises are: (i) Adverse Childhood Experiences (ACEs or potentially traumatic events experienced during infancy), such as violence victimization and forced displacement have devastating effects on ECD (Shonkoff and Phillips, 2000; Felitti, 2002; Walker et al., 2007); (ii) a healthy child–parent emotional bond can promote resilience and a proper ECD, even in contexts of extreme adversity (Lieberman et al., 2006; Ippen et al., 2011); and (iii) Exposure to traumatic experiences affects caregiver’s mental health and hinders their capacity to provide a secure and healthy attachment (Lieberman and Van Horn, 2011).

Given these premises, the program’s curriculum is structured to achieve the following objectives: (1) generate capacity for self-reflection and a non-judgmental curiosity about own and children’s internal emotional universe; (2) promote sensory integration and adopt self-regulation and stress management tools; (3) raise awareness on the capability of resilience that caregivers and their children have; (4) give new meaning to past traumatic experiences and restore trust; (5) give a new meaning to childrearing support networks and build nurturing teams; (6) strengthen the relationship between caregivers and children; and (7) Increase the repertoire of context-relevant and culturally appropriate parenting strategies. As proposed by the theory of change, if these objectives are achieved, in the short-term SA should: (I) improve caregiver’s mental health, (II) build trust and strengthen childrearing support network and nurturing team, and (III) promote a strong and healthy emotional bond between the child and their caregivers. In turn, these transformations are expected to support the socioemotional and cognitive development of the child in the long run.

*Semillas de Apego* builds upon the framework and structure of the Child–Parent Psychotherapy (CPP) (Lieberman and Van Horn, 2011) and Building Bridges programs (Reyes and Lieberman, 2010). The CPP is a clinical intervention with a multi-theory framework -including attachment, cognitive behavioral, developmental, psychodynamic, and trauma informed theories, among others- that has proven to be effective in improving the mental health and behavior of children, and strengthening child–parent relationships in households exposed to traumatizing events such as: domestic violence (Lieberman et al., 2006; Ippen et al., 2011), child maltreatment (Cicchetti et al., 1999; Toth et al., 2002, 2015), and caregivers struggling with depression (Cicchetti et al., 1999; Guild et al., 2017). Building Bridges is a non-clinical group-based intervention, also inspired by CPP, that has suggestive evidence on its relevance and scalability, but its impact has not been yet evaluated.

*Semillas de Apego* consists of 15 multi-caregiver group sessions, delivered once per week. Each session lasts approximately two and a half hours. Each group includes between 12 and 16 participants, all primary caregivers of at least one child 0–5 years old. Maintaining the same structure in all sessions and the same group composition makes the weekly meetings a predictable space for the participants, which generates a sense of safety and promotes trust and closeness among the group members. All the sessions have the following sequence of activities: a welcoming moment (to socialize, share important life events and talk about exercises left to practice at home during the week), a warm-up activity (to connect to emotional and physical state through mindfulness and body practices), a core activity (usually involving self-reflection and arts and crafts) and a closing moment (to share experiences and collect learnings from the session). The main objective of the core activities for each session is broadly described in Appendix A. SA uses a *task shifting* approach, which is a human resource management strategy originally developed to address public health crises in contexts where highly qualified professionals are scarce (Orkin et al., 2021). Specifically, task shifting aims at re-distributing tasks from highly qualified workers to workers with fewer qualifications to make more efficient use of the available human recourses (WHO, 2008). In the case of SA, all group sessions are led by two “facilitators,” who are community members, are caregivers themselves, and have participated in a six-week experiential training. Although the facilitators are not required to have prior experience nor formal training in psychosocial interventions, the selection process of SA does favor local community members who have some experience leading group activities. Facilitators have the responsibility of recruiting participants, delivering the program sessions, reporting monitoring data, engaging in reflective supervision, and handling the necessary logistics to successfully conduct the group sessions.

*Semillas de Apego* adapted the *reflective supervision* model from the one implemented in the CPP. The reflective supervision protocol in SA includes weekly face-to-face group meetings led by a “technical supervisor,” and individual meetings or calls between the same supervisor and each facilitator. These meetings are mandatory for all the facilitators. The reflective supervision protocol is an essential component of the program for two reasons. First, the meetings seek to deepen core elements of the intervention by discussing the progress of participants, following-up on at-risk cases, and revising technical and logistical issues. In addition to the monitoring system delivered through a digital platform (described in section 3.3.2), the reflective supervision protocol is a fundamental mechanism to monitor the quality of the sessions. Second, this protocol creates safe spaces to address personal matters that can potentially affect the facilitators and could thus have a negative impact on implementation quality. In addition to providing continuing technical support, the supervision protocol aims to mitigate occupational hazards that are common among professionals working in the mental health field (e.g., vicarious trauma, compassion fatigue, psychosocial distress, and burnout syndrome) (Susman-Stillman et al., 2020; Wilson et al., 2021; Barron et al., 2022).

The final role in the implementation team is the “general technical director,” who ensures there is a successful adaptation and implementation of SA to the context where it will be delivered, oversees all technical issues (throughout implementation processes), leads the reflective supervision for the team of supervisors, and intermittently accompanies reflective supervision sessions for the facilitators. Additionally, the director oversees strengthening and managing the network of local institutional allies that support the implementation of SA, and reports to the program’s management team about the progress and challenges in the program implementation.

During the 2018–2019 implementation phase, the program had the following support materials: the curriculum, a written document that presented the detailed the objectives each session, scripted all the activities in the session, described the needed arts, crafts and other materials; a training manual, which included the detailed workplan for the training of the facilitators and a summary of the theory supporting the design of the intervention; a technical guide, a written document summarizing theoretical and technical concepts related to the science supporting the program; and a simple guide for basic breath technics and mindfulness practices.

### 2.2. Context: Tumaco

Tumaco is a 200,000-inhabitant municipality that lies in the Pacific coast of Colombia, in the border with Ecuador. Historically, it has been a setting extremely affected by violence and poverty. In 2017, 243 homicides were reported in Tumaco, representing a rate per 100,000 inhabitants of 116.6. These violence figures exceed those recorded in the most violent cities in the world -such as Los Cabos (111.3), Caracas (111.2), and Acapulco (106.6) (Ortega, 2018)-, and is more than 10 times higher than those observed in many countries amid active civil conflict, −such as Afghanistan (6.5), Iraq (8.0), Somalia (8.0), and South Sudan (13.9) (UNODC, 2019). The fact that this municipality has the largest number of illicit crops in the country (23,148 hectares), half of its population lives with unsatisfied basic needs, 23% of the working-age population are unemployed and 92% of the employed population work in the informal sector, reveals several of the structural determinants of violence in Tumaco (UNODC, 2019; DANE, 2011; UNICEF, 2017).

### 2.3. Study design

In 2015, the team led a pilot study of SA in Bogotá with 64 participants (divided into three groups), all of them victims of forced displacement. The pilot, funded by the Ministry of Health and Social Protection (MoH) and the Interamerican Development Bank (IADB), was the first implementation of the SA curriculum and included a process evaluation and a results evaluation. This implementation phase was the result of a collaboration with the Mayor’s Office of Bogotá that aimed at integrating SA to the service portfolio offered to internally displaced families in the city. The pilot provided evidence that suggested the validity and appropriateness of the SA curriculum (see Harker Roa et al., 2017).

To further strengthen the program in the face of a future scale-up phase by identifying the program’s impact and implementation enablers and barriers, between 2018 and 2019 a process evaluation and an impact evaluation (Moya et al., 2022) were carried out simultaneously. The process evaluation, which is the focus of this article, concentrated on seven measures to assess implementation quality: (i) fidelity, (ii) acceptability, (iii) adoption, (iv) appropriateness, (v) feasibility, (vi) penetration, and (vii) sustainability (Proctor et al., 2011; Smith et al., 2018). These constructs are widely used to define implementation outcomes for mental health and behavioral interventions (Proctor et al., 2009; Lewis et al., 2015a,b). To gather information on the process evaluation, qualitative and quantitative data were gathered from different sources (Table 1). All the procedures were approved by the Institutional Review Board of Universidad de los Andes (record #1303, February 2021).

**Table 1:** Measures of “implementation quality” and information source.

Construct	Definition	Key Informant			Monitoring System
		**Technical Team**	**Program facilitators**	**Program Participants**	
Acceptability	“The perception among implementation stakeholders that a given treatment, service, practice, or innovation is agreeable, palatable, or satisfactory” (Proctor et al., 2011, p. 67)	No	No	Yes	No
Adoption	“The intention, initial decision, or action to try or employ an innovation or evidence-based practice” (Idem, p. 69)	No	No	Yes	No
Appropriateness	“The perceived fit, relevance, or compatibility of the innovation or evidence-based practice for a given practice setting, provider, or consumer; and/or perceived fit of the innovation to address a particular issue or problem” (Idem, p. 69)	Yes	Yes	Yes	No
Feasibility	“The extent to which a new treatment, or an innovation, can be successfully used or carried out within a given agency or setting” (Idem, p. 69)	Yes	Yes	No	No
Fidelity	“The degree to which an intervention was implemented as it was prescribed in the original protocol or as it was intended by the program developers.” (Idem, p. 69)	Yes	Yes	No	Yes
Penetration	“The integration of a practice within a service setting and its subsystems” (Idem, p. 70)	Yes	Yes	No	No
Sustainability	“The extent to which a newly implemented treatment is maintained or institutionalized within a service setting’s ongoing, stable operations” (Idem, p. 70)	Yes	No	No	No

Source: Excerpts taken from Proctor et al. (2011).

#### 2.3.1. Qualitative approach

Qualitative data was collected through Key Informant Interviews (KIIs) to explore the proposed research questions (see section 1). KIIs were guided by the *Phenomenological Interviewing* framework, aiming at understanding the factors associated with implementation success (i.e., the phenomenon of interest), through the testimonies of actors who had first-hand experiences in the program (Englander, 2012). Semi-structured interview guides were designed for each of the three groups of key informants included in the field work. The first group was the technical team of SA, which included three professionals: the Technical Consultant (affiliated to the University of California San Francisco), the General Technical Director (affiliated to SA) and Technical Supervisors (affiliated to SA, *N* = 2). The second group of key informants were the team of program facilitators (*N* = 6), which was made up by community agents who were affiliated to Genesis Foundation, the NGO implementing the program. Interviews with members of these two groups aimed at collecting information on general implementation challenges, and focused on the appropriateness, feasibility, and potential penetration of SA. Finally, the third group included a sample of primary caregivers (*N* = 9; 8 females, 1 male) that participated in the program. The interviews were designed to collect information about the implementation quality of the program. The interviews also explored the appropriateness, acceptability and adoption of the psychosocial and caregiving strategies discussed throughout the sessions. Each one of the interview guides explored the constructs described in Table 1. Examples of the questions used are presented in Table 2.

**Table 2:** Example questions to explore each construct of implementation quality.

Construct	Questions
Acceptability	How satisfied are you with the information and content of Semillas de Apego? What did you like about the program? What would have you liked to be different [regarding the program]?
Adoption	Can you give us an example of something you learned in the program and how you use it with your family/children? What has motivated you to make it a habit or daily practice?
Appropriateness	What makes the Semillas de Apego program suitable for the reality of Tumaco?
Feasibility	In your opinion, what allows Semillas de Apego to be successfully implemented in Tumaco? What does the program have that makes it possible to be implemented in Tumaco?
Fidelity	In your own words, what does Semillas de Apego aim to achieve? What did the trainings consist of? How often were they [the trainings] carried out? How do you think these trainings help you in your role as facilitator? What trainings help you the most?
Penetration	How does the economic incentive help your participation in the program? How can Semillas the Apego integrate to local or government institutional service settings?
Sustainability	What would you say are the biggest challenges that Semillas de Apego faces today? What would help you do your job better? What are your recommendations for the future expansion of the program?

Questions translated to English from the KII guides.

All interviews were conducted between December 2020 and February 2021. The interviews were recorded, transcribed by research assistants, organized and systematized using Nvivo Software®. The process of data analysis was done in two phases. Initially, the interviews were grouped together based on the key actor represented in each interview and were analyzed accordingly. Each question asked during the interviews was classified based on the constructs from the interview guide (Table 1). In the second phase, the information that was previously sorted into the different constructs was coded. Three coders were trained to code the data. Before they started coding, a training session on open coding was led by one of the co-authors (NC). Open coding activities were then used to break down the data into emergent categories, which were then used to classify the information within each construct (see Table 3) (Strauss, 1990; Strauss and Corbin, 2002; Nathaniel, 2021).

**Table 3:** Emergent categories.

Construct	Emergent categories
Fidelity	- Strong process of recruitment, selection, and training of facilitators
	- Continuous support of facilitators
Appropriateness	- Flexibility for adaptation to context and culture
Acceptability	- Relative importance of subsidies
Adoption	- Positive parenting
	- New support networks
	- Mindful breathing
Sustainability	- Security
	- Local support and commitment
	- Funding

#### 2.3.2. Quantitative approach

The quantitative research design focuses on leveraging evidence on the fidelity of the implementation, defined as the extent to which SA was executed in Tumaco as it was prescribed in the original protocol (Dusenbury et al., 2003). As explained by Proctor et al. (2011), fidelity is measured “typically by comparing the original evidence based intervention and the disseminated/implemented intervention in terms of (1) adherence to the program protocol, (2) dose or amount of program delivered, and (3) quality of program delivery” (p. 70). Specifically, in this study the quantitative approach provides measures of dosage, but not of adherence -defined as the degree to which the sessions occurred as intended (Hogue et al., 1996)- or the quality of the delivery.

The quantitative information used in this study was collected through a monitoring system designed for SA, which is structured and delivered through the digital platform KoboToolbox®. The system is comprised of custom-made digital forms that collect information provided by the program facilitators, after the execution of each of the 15 sessions in the program’s curriculum. Each form includes 4 to 5 open- and close-ended items that inquire about: achievement of goals or milestones in each weekly session and logistics (i.e., availability and quality of spaces, materials, among others). All program facilitators were trained and initially received weekly support to use the digital platform.

At this stage, the monitoring system was able only to consistently collect information on participant attendance. Recurrent structural changes made to the monitoring system during the 2018–2019 implementation phase made it impossible to create indicators of adherence and quality of program delivery, across time and cohorts. Therefore, the quantitative analysis in this process evaluation study focuses on the degree to which SA was implemented as it was prescribed in the original protocol (i.e., fidelity) in terms of the number of sessions delivered to each participant.

In addition to descriptive analyses of the proposed dosage measures, a survival analysis (see Allison, 1984) was conducted using participant attendance data to identify the factors associated with the probability of participating in a “sufficient” number sessions - or, alternatively, the probability of not dropping out. To do this, a *time of survival* measure (“t”) was created based on the information on the last session attended by each participant before either dropping out or successfully graduating from the program. A participant who continued in SA until session 15 was assigned a value of 15 (*t* = 15), while a participant who did not return to the program after session 6, was assigned a value of 6 (*t* = 6). Afterwards, two different dropout indicators were created using this *time of survival* measure. The first indicator, denominated “observed dropout,” is solely based on being present until the end of the program: all individuals who did not attend the 15th session are considered to have dropped out of the program. The second dropout indicator, the “normative dropout” measure, is based on a “minimum dose” that a person should receive in order to expect SA to have a significant impact. Thus, all individuals who did not attend at least 12 sessions are considered to have dropped out of the program. In the design phase, this threshold was defined by the technical team and is formally considered an “expulsion rule” in the set of norms of SA. Moreover, before program initiation, all potential participants are forewarned that if they miss three or more sessions, they will be asked to leave the program. Also, to capitalize the trust-building activities that are at the core of the initial weeks of SA, participants are cautioned that they would also be asked to leave if they miss more than one of the first 3 sessions of the program.

The objective of the survival analysis is to estimate the hazard ratios (HR) across different “participant profiles” using a Cox Proportional Hazards Model (Allison, 2014). To create participant profiles, the data from the monitoring system was merged with data collected in the baseline survey of the impact evaluation study of SA (see Moya et al., 2022), which includes characteristics of the caregiver (i.e., the program participant), her household (family and dwelling) and her child (details in Section 3). The initial raw sample for this analysis included a total of 712 participants, all who were enrolled to the program and participated in the baseline survey. After excluding observations with missing values and outliers, our analytical sample includes 647 observations (90.9% of the total participants enrolled).

## 3. Results

The results of the process evaluation study aimed to identify information around seven spheres, namely: acceptability, adoption, appropriateness, feasibility, fidelity, penetration, and sustainability (Proctor et al., 2011; Smith et al., 2018). However, the evidence produced by the qualitative and quantitative analyses was not robust enough to support conclusions related to the “feasibility” and “penetration” dimensions. The themes that arose from the qualitative data analysis were particularly related to the spheres of acceptability, adoption, appropriateness, fidelity and sustainability. The results from the quantitative analysis focused only on measures related to the fidelity dimension. This section presents qualitative and quantitative evidence of factors that enabled the implementation of SA, which could eventually inform the development and expansion of psychosocial support interventions with primary caregivers to promote ECD in contexts impacted by violence and forced displacement.

### 3.1. Fidelity

Evidence on the fidelity in this phase of implementation (2018–2019) of SA comes from both the quantitative and qualitative analyses. On the one hand, the quantitative approach provides evidence on the dosage (i.e., the number of sessions delivered) and the factors associated with a higher probability of compliance (i.e., participating in enough sessions to perceive the benefits of the intervention). On the other hand, results from the qualitative approach relate to the level of adherence to the protocol and the quality of the delivery. There were two mechanisms to monitor adherence and quality of delivery in the implementation phase this study evaluates. The first one was the monitoring system. The second mechanism was the *reflective supervision* protocol. The qualitative information collected provides evidence on the successful implementation of processes that play a key role to guarantee adherence and quality in the delivery (as explained in section 2): (i) recruitment, selection, and training of program facilitators; and (ii) the continuous support of facilitators.

#### 3.1.1. Dosage measures and trends

Table 4 presents, separately for each of the four cohorts implemented in Tumaco during 2018–2019, the count of persons invited to participate in the program, the count of persons that enrolled, and the count of persons that attended to at least one session – which we define as an “initial take-up.” Using information collected in the monitoring system, we constructed three measures of dosage. The first measure is the enrollment rate, which shows the proportion of caregivers that, after receiving a general description of the program and a formal invitation to participate, confirmed their interest to join the 4-month program and participated in a baseline survey. On average, 63% of the persons invited made an initial commitment to participate. This proportion was relatively homogenous across cohorts and oscillated between 60% (cohort 4) and 65% (cohort 3).

**Table 4:** Take-up and adherence measures, across cohorts.

		Cohort				Total
		1	2	3	4	
[1]	Invited	215	238	362	324	1,139
[2]	Enrolled	132	151	237	193	713
[3]	Initial take-up^*^	91	117	187	154	549
[4]	Enrollment rate ([2]/[1])	61%	63%	65%	60%	63%
[5]	Take-up rate ([3]/[2])	69%	77%	79%	80%	77%
[6]	Avg. sessions attended (after take-up)^+^	7.6	11.9	11.0	11.6	10.8

Constructed using administrative ledgers of SA. *Attended to at least one session. ^+^Average number of sessions attended by all caregivers that had an initial take-up (i.e., persons who were invited, then enrolled and finally attended to at least one session).

The second measure, the take-up rate, is the proportion of the initially committed caregivers that attended to at least one session. For the total 2018–2019 implementation phase in Tumaco, 77% of the persons that enrolled, had an initial take-up of the program. An important result is that this proportion increased significantly, suggesting that important implementation barriers were surpassed from the second cohort onwards. This result is confirmed by the trajectory of the third dosage measure, the average number of sessions attended (after take-up): for the first cohort the average number of sessions was 7.6, while for the second, third and fourth it was 11.9, 11.0, 11.6, and 10.8, respectively. At an ideal level of fidelity (in terms of dosage) -that is, when SA is implemented as it was prescribed in the original protocol-, take-up rates would be 100% and the average number of sessions attended would be 15.

**Figure 2:**
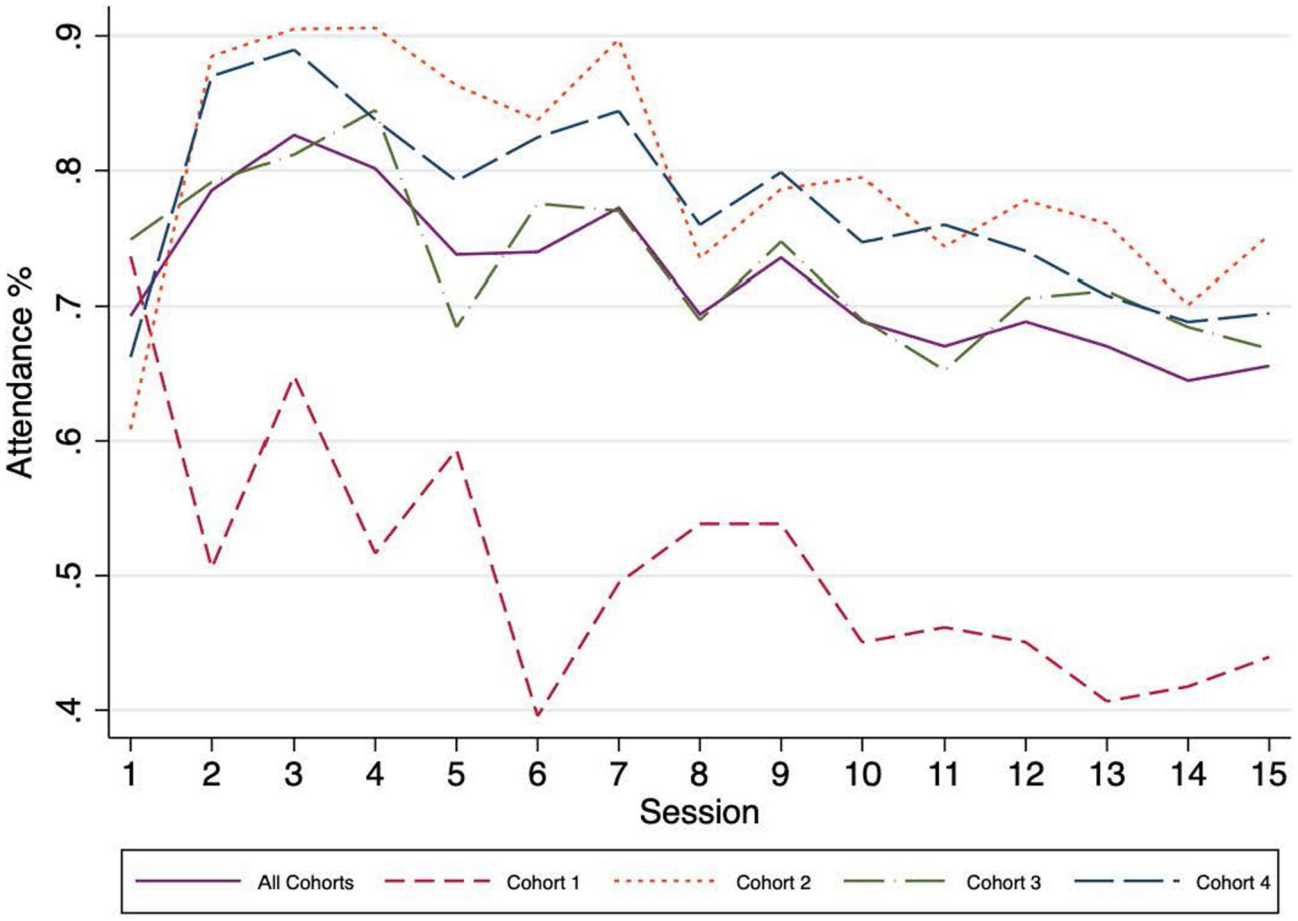
Attendance rates after initial take-up, by session and cohort.

Figure 2 presents the average attendance rate (the third dosage measure), by session and cohort, for the caregivers that had an initial take-up. At least three messages come up from the graph. First, there is a negative time gradient: attendance rates drop as the sessions advance, in all cohorts. Second, while the attendance rate for the first cohort is above 60% for only two sessions, for the subsequent cohorts most of the sessions had attendance rates are above 70%. Third, there is a high volatility in attendance rates, within and across cohorts: maximum attendance rate levels are observed in sessions 2, 3, and 4 (above 90% for cohort 4), and minimum levels are observed in sessions 14 and 15 (around 45%, for cohort 1). To better understand the determinants behind this variation on the level of fidelity in terms of dosage, a survival analysis is presented in the next section.

#### 3.1.2. Determinants of non-compliance

As mentioned before, attendance registries were used to construct two proxy measures of non-compliance: “*observed* dropouts” and “*normative* dropouts.” The distribution of these two indicators is used to capture the average level and variation of fidelity -i.e., how close was the implementation to the original protocol’s prescription in terms of the amount of program delivered or dosage-, overall and across participant profiles (described by the set of observable characteristics described in Table 5). Participants in the study (98% woman) are extremely vulnerable: only 11% have formal employment, 50% report no monthly labor income, 75% have at most secondary education, 59% report having been forcefully displaced and 84% being direct victims of violence (see Table 5). In addition, 40% of the households do not have access to public water supply or sewage service, 43% are beneficiaries of a conditional cash transfer program, and 28% are mono-parental. It is important to highlight that there is still an important heterogeneity in the severity of all sources of vulnerability, at the participant and the household level. We observe that most of the abovementioned variables have a relatively large coefficient of variation (defined as the relative magnitude of the standard deviation relative to the mean). This is an important result given that it provides an opportunity to explore potential determinants of the amount of program delivered to a participant, which is a proxy of fidelity.

**Table 5:** Descriptive statistics for the variables included in the survival analysis.

	*N*	Mean	SD	Min	Max	P25	P50	P75
Dosage measures								
Time of survival (t)	647	8.43	6.05	0	15	1	12	14
Normative dropout (=1)	647	0.54	0.50	0	1	0	1	1
Observed dropout (=1)	647	0.49	0.50	0	1	0	0	1
Attended at least 2 of first 3 (=1)	647	0.64	0.48	0	1	0	1	1
Caregiver characteristics								
SA Cohort (1, 2, 3, or 4)	647	2.74	1.04	1	4	2	3	4
Caregiver’s age	647	28.94	9.51	16	78	22	26	33
Caregiver is female (=1)	647	0.98	0.14	0	1	1	1	1
Monthly labor income (USD)	647	199	280	0	1,177	0	0	313
Caregiver is formal worker (=1)	647	0.11	0.31	0	1	0	0	0
Caregiver’s education level (1, 2, or 3)	647	1.07	0.64	0	2	1	1	1
Caregiver is child’s parent (=1)	647	0.91	0.28	0	1	1	1	1
Severity index > risk threshold (=1)	647	0.21	0.41	0	1	0	0	0
Caregiver is IDP (=1)	647	0.59	0.49	0	1	0	1	1
Caregiver is violence victim (=1)	647	0.84	0.37	0	1	1	1	1
Household characteristics								
Number of children under 5	647	1.32	0.61	1	5	1	1	2
Two parent household (=1)	647	0.72	0.45	0	1	0	1	1
Household asset index	647	−1.64	1.18	−4.90	2.87	−2.42	−1.63	−0.82
Access to water and sanitation (=1)	647	0.60	0.49	0	1	0	1	1
CCT beneficiary (=1)	647	0.43	0.50	0	1	0	0	1
Child characteristics								
Child’s age	647	2.55	0.72	1	5	2	2	3

Impact evaluation study baseline survey (Moya et al., 2022) and SA monitoring system. “Cohort” indicates the cohort of the program for each participant. Caregiver’s education level: = 0 if the participant has primary education or less, = 1 if the participant has completed or some secondary education, and =2 if the participant has completed or some tertiary education. Severity index above risk threshold: 1 if the severity index is above the risk threshold. Caregiver is an internally displaced person: = 1 if the participant has suffered from internal displacement (= 0, otherwise). Caregiver is victim of direct violence: = 1 if the participant has been a victim of direct violence (= 0, otherwise). Number of children under 5 years: is the count of children under 5 years old who live in the household. Two parent household: = 1 if the household is composed of two parents (= 0, otherwise). Household asset index: Standardized measure of structural wealth based on self-reported ownership of assets.

According to the time of survival indicator, of the 647 participants in the analytical sample 25% attended to only the 1st session, 50% reached the 12th session, and 75% reached the 14th session. This pattern is replicated by the two non-compliance indicators: 49% did not reach the program’s 15th session (thus classified as “observed dropouts”), and 54% did not attend at least 12 of the 15 sessions (“normative dropouts”).

Table 6 presents the estimated hazard ratios (HRs) for the two proposed Cox Proportional Hazards models. Column 1 presents estimates for the model that predicts “observed dropout” events and Column 2 for the model that predicts “normative dropout” events. In its rows, the table presents the estimated HR across two groups (or profiles) defined by the dichotomous explanatory variable in the row and the standard error for this HR below (in parenthesis). The results show that, after controlling for the full set of covariates included in the models, the following characteristics reduce the probability of not reaching the 15th session (and thus improve the level of fidelity): attending to at least 2 of the first 3 sessions (93.2% lower for this group), female participants (72.4% lower), and having secondary or tertiary education (45.5 and 44.5% lower, respectively). On the contrary, “observed dropout” probability increases if the caregiver is employed in the formal sector (195% higher) and if she is a direct victim of violence (66.1% higher). Similar results -in terms of the sign and at the same level of statistical significance of the HRs- are obtained when the predicted event is the “normative dropout” (see column 2, Table 6).

**Table 6:** Descriptive statistics for the variables included in the survival analysis.

Predicted variable	Observed dropout (1)	Normative dropout (2)
Caregiver characteristics		
Cohort 2 [=1]	0.743	0.823
	(0.353)	(0.389)
Cohort 3 [=1]	1.654	2.287**
	(0.665)	(0.878)
Cohort 4 [=1]	0.714	0.969
	(0.351)	(0.430)
Attended at least 2 of the first 3 sessions [=1]	0.068***	0.100***
	(0.024)	(0.030)
Caregiver’s age	0.948	0.968
	(0.046)	(0.040)
Caregiver’s age squared	1.000	1.000
	(0.001)	(0.001)
Caregiver is female [=1]	0.276***	0.258***
	(0.110)	(0.099)
Caregiver’s income (log)	0.830	0.831
	(0.129)	(0.121)
Caregiver’s income squared (log)	1.026	1.024
	(0.023)	(0.022)
Caregiver is formal worker [=1]	2.951***	1.425
	(1.138)	(0.351)
Secondary Education [=1]	0.545**	0.354***
	(0.130)	(0.136)
Tertiary Education [=1]	0.555**	0.593
	(0.153)	(0.245)
Caregiver is the child’s parent [=1]	1.370	1.031
	(0.596)	(0.321)
Severity index above mental health risk threshold [=1]	1.172	1.251
	(0.223)	(0.211)
Internally displaced person [=1]	1.139	1.169
	(0.214)	(0.195)
Victim of direct violence [=1]	1.661**	1.453*
	(0.423)	(0.314)
Household characteristics		
Number of children under 5 years	0.841	0.791*
	(0.109)	(0.095)
Two parent household [=1]	0.999	0.991
	(0.186)	(0.174)
Household asset index	1.122	1.150*
	(0.093)	(0.087)
Access to public water supply or sewage [=1]	0.805	0.903	Predicted variable	Observed dropout (1)	Normative dropout (2)
	(0.163)	(0.169)
CCT beneficiary [=1]	1.230	1.330*
	(0.209)	(0.211)
Child characteristics		
Child’s age	1.046	1.060
	(0.109)	(0.102)
Observations	501	501

Exponentiated coefficients. Robust standard errors in parenthesis. Specification estimated through Cox Proportional-Hazards Model. A formal statistical test for the “proportional hazards” assumption was conducted using Schoenfeld residuals. Given that the evidence that some covariates were time-varying and thus the original model violated the assumption, interaction terms between these time-varying covariates and the time of survival variable were included in the final model. Also, as a robustness check the models were estimated using a rescaled version of the time of survival variable (by adding 1 to each t). *p < 0.1, **p < 0.05, ***p < 0.01.

#### 3.1.3. Strong process of recruitment, selection, and training of facilitators

In the interviews with the technical team, they indicated that the recruitment and selection of facilitators was one of the main steps to ensure an effective training and the successful implementation of SA. The technical team agreed on three key factors to achieve a solid team: (i) doing face-to-face interviews with applicants, (ii) prioritizing the willingness of the candidates to deal with emotional and therapeutic processes and (iii) selecting persons that demonstrate honesty and transparency. One of the members of the technical team mentioned:

*“We decided to have a selection process. We held a group session where we provided them with a first experience of what the selection process would be like. This taught us a great deal! We received resumes that, given their academic training, made them seem as though they were the right fit. But we ended up choosing more intuitively and not according to the resumes, in favor of people whom we truly felt would be able to deal with the process*”*. BN Technical Team*

According to the facilitators interviewed, the training for the program went beyond the initial training sessions and it implied constant learning. In fact, the training sessions and program reviews were recurrent; they took place once a week and included on-site visits by the team of supervisors, every 15 days. The team’s perception of the training is one of constant support and availability from the supervision team. Regarding training, the facilitators mentioned:

*“I feel that throughout all these years we have been constantly training because we never stop learning, there’s always something new. The people who have been in charge have always been very much focused on us as facilitators making use of all the tools so that we leave nothing behind.” Facilitator O*

*“It’s wonderful, that we are not alone. We are always accompanied by them, by our supervisor, our coordinator. If something happens all of a sudden, they are around 24-7 and will always give us the support we need. I think we haven’t been left there, abandoned, they always give us their support”. Facilitator V*

The facilitators also mentioned that one of the most effective learning experiences was participating in the program itself, as women and as mothers, before facilitating the sessions. In other words, the facilitators agreed that it was important to understand the program based on their own processes of childrearing and life experiences (frequently marked by violence, abuse, and trauma). This led the facilitators and participants in the program to establish relationships of empathy and trust, and to use the experience of the facilitators as real-life examples that can be achieved thanks to the processes proposed in SA. The facilitators referred only to the experiential processes as the most appropriate for achieving good implementation.

*“For example, in the activities that we carry out during the training, we include abuse, which is what I personally worked on with my son. I can now let go, speak, and recognize what I did with my son, which helps me to help mothers who will be entering the process, so that I can say: ‘Look, you can overcome this, you can transform this maltreatment into something positive for your child, in strengthening these relationships.’ Why? Because I went through this myself, I did it”. Facilitator*

*“So, during the training, this could allow me to acknowledge my mistakes and where I am failing as a mother; this is a very specific tool; that helps mothers be open to this relationship, and they begin to believe in the facilitator. That is one of the marvelous touches of Semillas, the fact that they can see the facilitator as an average person who this also happens to and who has also gone through what they are experiencing. We do not judge them, nor do we look at them as if to say: ‘you are the worst mother*’.” *Facilitator*

“*Our script is rigorous, but there are parts where you must provide an example from your personal life. We tell them: ‘I also beat them, I also did such and such, I was also violent, I did this...’. So, the mother feels that the person who is speaking is human, that we are connected.*” *Facilitator V*

#### 3.1.4. Continuous support of facilitators

As mentioned before, SA adopted the reflective supervision model which provides a structure for the continuous training process of the facilitating team. Through individual and group supervision activities, facilitators received feedback from the technical team regarding the fidelity of the implementation of the curriculum, in terms of the adherence to the protocol and the quality of the delivery. According to the technical team, this reflective supervision takes place regularly and it considers the relationships between supervisors and professionals, between professionals and caregivers, and finally between caregivers and their children. A member of the technical team summarized the continuous support in the following way:

“*In their first trip, the primary team began with the pilot project in Bogotá. It then continued its support for the team in this context, which we call reflexive supervision, in which they deal with clinical dilemmas regarding how to apply the model for different circumstances or groups and they are worked on collaboratively.*” *VR Technical Team*

*“We think that this type of reflection and supervision, which is reflective, creates emotional support spaces that become part of a parallel process. And I think that they are critical in this type of work, because it evokes a lot in a person. One sees a lot of traumas, poverty, injustice. [...] We think that you need someone a bit more distanced from the system who can help you think about what you can come and say, ‘Oh, I feel this way!’. And you can receive this type of support”. VR Technical Team*

As stated by the technical team, *reflective supervision* is conceived to provide *technical support*, but also as a strategy of *psycho-emotional well-being* for professionals who work with families and children in highly adverse contexts. According to the interviews, this type of supervision enables the team to manage strong emotions and carry out reflexive actions to tailor the implementation practices. The facilitators discussed at great length the benefits of reflective supervision by stating:

*“Reflective group supervision is about how to understand or ask how the group is doing. And when a particular circumstance, situation or weakness is generated, it must be strengthened through exercises, breathing, workshops, or homework that they assign to us. So, it’s wonderful to see how we are growing little by little and strengthen things that we were perhaps not so strong in, even theoretical things.” Facilitator M*

*“Well, at the individual level, we talk about more personal and deeper matters that sometimes affect both the work and the person. So, it is more a matter of getting deeper into personal matters, asking: ‘how do we feel? Did it help the session run smoothly? Did it prevent us from carrying out the session […]? What is happening in our lives at that moment?’ That kind of things.” Facilitator O*

The interviews with the facilitators further revealed that reflective supervision is a model that favors professional well-being and allows the team to feel recognized from the emotional perspective, which helps them not only to deliver the sessions with the expected quality but also to improve their responses to conflict in their family and daily lives.

*“I have never had this kind of attention towards myself. Do you understand? It was also a matter of picking up the form, going out there, applying and taking it and getting things done. They are concerned about us here, how we are doing, our relationships with our colleagues, bosses, children, family; it’s comprehensive. To me this is a beautiful aspect of the program and hopefully it will never change, the end goal, of not just dealing with the community or the mothers, or the people that we’ll be working with, the children, early childhood, but also with the facilitator who can also recharge and be nourished by the experiences projected by the community.” Facilitator O*

*“One of the training sessions was very intense. It focused on [past] trauma. The things I carried with me from childhood, and they made me see that the world can be different. And they made me start to change patterns that I had formed in my childhood, negative things, things that are not good for me. And the therapy that they performed on us during the training sessions helped me a great deal. I had fallen into a very similar pattern to my mother’s. It was not a positive thing, it was negative. And I really understood that we can change those child-rearing patterns and be different. I do not want to be like my mother, I want to be a different person. And that was one of the things for which I am most grateful to the supervisor and to the program, because I really cut out that pattern and now, I am another person, with different qualities. This left a mark on me and helps me when I am in the field, and I am working with my group. To speak to them based on my own example, the experiences I had and went through to transmit it to them.” Facilitator G*

### 3.2. Appropriateness

Appropriateness refers to the relevance the intervention has among providers and/or participants (Proctor et al., 2011). In this particular study, appropriateness was brought up several times by the facilitators and the technical team and it focused specifically on the flexibility of the curriculum and the subtle, yet meaningful changes that needed to be made to adapt it to the specific conditions of Tumaco.

#### 3.2.1. Flexibility for adaptation to context and culture

Through the interviews with facilitators, it became evident that the SA curriculum allows for flexibility to recognize and integrate the contextual and cultural factors of the community within which the program is being implemented. Although the program’s curriculum has core components that should not be modified, the implementation protocol of SA explicitly recognizes that certain components must be adapted to the local context. This adaptation processes involves collaborating with community agents and recognizing their knowledge and understanding the social, economic, cultural, and political realities of the context. It was also necessary to consider different pedagogical strategies in order to teach the content of the manual to the facilitators. In the case of Tumaco, the technical team mentioned that an “experiential learning” methodology was necessary to effectively train the local team of program facilitators. The team arrived at this approach after an initial -and mostly unsuccessful- attempt to do the training based on autonomous reading of the program’s manual and additional supporting lectures. Members of the technical team mentioned:

*“The team (in Tumaco), for example, had not read the curriculum. This was a challenge that we faced in the beginning because there was a cultural difference in the context of Tumaco. […] We decided in our consultation that instead of forcing our way of working, which mostly comes from Bogotá, why don’t we learn to work as they do? Maybe it’s more organic and maybe it’s not a matter of reading. Since they are not going to read what is assigned to them, what if change our methodology to match theirs? What if we go at their pace instead of imposing our own? We should not claim that they are not paying attention or that they don’t care, when in reality, something else is happening there.” VR Technical Team*

*“The arrival of Semillas de Apego in Tumaco involved various trial and error exercises through which the technical team reached the conclusion that the team should be trained in an experiential manner. The importance of living the program, of incorporating the tools in the day-to-day lives of the facilitators, was consolidated as one of the criteria for training the local teams on the theoretical contents and group management and including it as a factor to consider in training for adults” BN Technical Team*

Facilitators also agreed that acknowledging cultural differences was crucial for the delivery of SA in Tumaco. Most cultural changes had to do with the integration of colloquial language that people could easily understand. But also, the cultural adaptation implied a consideration on how the traditions of the Colombian Pacific could be integrated to the curriculum to relate its content and objectives to the culture of the participants. For instance, oral traditions of Afro-Colombians were integrated through children’s songs and lullabies that are very present in local child-rearing practices (Meneses Copete, 2022). One of the facilitators summarized this by saying:

*“The first part was the most difficult. It involved dealing with this new type of work. So, it was about the approach and how to reach this community. Because sometimes it is not just a matter of looking at how the script [in the curriculum] is structured. No, you must speak as they do there, using colloquialisms, words that are spoken in my community and that we are familiar with. Because if you use very technical words in the mother’s group, they will not understand you. So, it is better to follow the script but to change that ‘particular word.’ The way we communicate among ourselves here in our territory and bring it down to that level so that they can understand. Because there are questions in some parts of the script where, if you ask the mothers the way it is written in the script, they will not understand. But we know how to change it, so that it will end up being the same question, but using words spoken here in this environment.” Facilitator G*

Also, adaptations to the context included changes in the music and materials used during arts and crafts workshops. The implementation team incorporated traditional instruments and music from Tumaco, which generated engagement and familiarity in the participants. Moreover, in an effort to reduce costs and environmental impacts, the implementation team incorporated materials endemic to the region to substitute the ones that had to be imported from other places.

*“We have done many things to adapt the program to the context, above all regarding the music that is played during the sessions, which we have been improving. We have been including things that are originally from the Pacific in the topics of the sessions. Things such as materials, or things like that, that can be obtained here, that we have been including, and this has also been helpful”. Facilitator O*

### 3.3. Acceptability

Interviews with participants provided important evidence on the acceptability of SA, in terms of how satisfied the caregivers were in the program, and if they perceived that the curriculum addressed their needs. For instance, caregivers highlighted how the program helped them to increase their capacity to regulate their emotions, understand the developmental needs of their children and increase the ability to interact positively with them. Participants mentioned:

*"It was a very good program, one that helps moms -not only new moms- to try to control their anger. Because all of us can suddenly become desperate. One has to be honest; it happens to everybody. But to control your breathing and say ‘well, just breathe’”. Participant A.*

*“It’s not that I'm the most explosive person. But [the program] taught you to control yourself, to calm down when the children are getting frantic, and to correct them with patience and to be a little more tolerant”. Participant I.*

#### 3.3.1. Relative importance of cash subsidies

Through our data collected from mothers, fathers, and caregivers, it became clear that although the incentives to attend the sessions were motivating - as participants could buy food and necessary items for their children - their main motivation were the benefits obtained from the program. We believe this is evidence that also supports the acceptability of the program. Program participants frequently mentioned this:

*“Yes, there was an incentive. They gave me 20,000 pesos [$4 USD] for each session. It [the money] was for the girl or whatever I wanted. […] But for me, the most important thing was not the money, but the learning received from this type of training, right? The most important thing for me was the training." Participant B*

*“I went mostly to receive the talks, which taught us useful things. But the money was also useful because I went to the supermarket and bought something for the kid or whatever I needed. As a single mother, everything helps me.” Participant F*

### 3.4. Adoption

Through the interviews, participants also shared how they integrated into their lives tools and strategies provided by SA. The adoption of self-care practices and parenting practices strongly suggests not only that the program is relevant for the context, but also that it can be successfully implemented.

#### 3.4.1. Parenting strategies and practices

In particular, frequently caregivers explained how their intention to change their way of interacting with their children is a consequence of them understanding the potential positive impact this has on the behavior, wellbeing, and development of their children.

*“It is also important to place ourselves in the child’s perspective and see how we can educate them in a way that does not affect their development. They are in that stage of development, and they are discovering everything. But we also must instill in them what is good and what is bad. Because why are we going to lie and say ‘this is good’, when it is bad […]. My girl, she likes the phone a lot, so I told my husband not to lend her the phone so much because every day she asks ‘mommy the phone’. She wants to be stuck watching videos of dolls all the time. So, I had to control those things more and try to teach them other types of games”. Participant E*

*“Sitting down to talk and then use the techniques they gave us there [in the program]. We are adults, obviously we are tall... but sitting or squatting down to’he child's height so that the child looks at us in the same position […] at that moment, we can talk in front of’he child's eyes and tell the child what is wrong and why should correct it. Then she will understand […]”. Participant O*

Participating in SA also helped to expand the caregivers’ comprehension regarding child development and to prioritize their needs as individuals. A participant mother mentioned:

*“There were many things that I ‘skipped’ or did my own way, because I thought that was the way to do it. In the program, they taught us how things should be in raising children. Because they are a reflection of bad parenting. [Before SA] my son, when he ate, would induce vomiting. And they explained to me that this happened if I forced him to eat and suddenly yelled at him, attacked him: 'eat, eat, eat' - it was something that the child did because I attacked him. All of that helped me with my kid. I was suffocating him too much at mealtime.” Participant C*

#### 3.4.2. Support networks

Additionally, program participants highlighted, thanks to the program, they were willing to share and create new spaces to support peers, thus creating new networks. Essentially, the participants expressed that they were open to build new relationships in their communities as a consequence of their participation in SA.

*Well, uh, sharing! Sharing with others, talking to others. Being more of a neighbor to my neighbor, being more attentive to my neighbor, who may be living in a worse situation than I am, and putting into practice what Semillas de Apego taught me” Participant A*

*"We shared a lot among all those who went to the meetings. But more than anything, we got to know each other." Participant G.*

#### 3.4.3. Mindful breathing

According to the interviews with participants, the activities related to mindfulness were the most memorable aspect of the program. Participants mentioned that mindful breathing exercises were particularly effective for managing stress and promoting relaxation. The participants highly valued practicing mindful breathing and recognized that this skill helped them improve their parenting skills and the communication with their children.

*“Well, the truth is, one of the things is learning to breathe, right? When you feel, you are going to explode! So, you go - think about it before doing it. You think and breathe, and it calms you down!” Participant G*

*“The breathing part. Learning to breathe. Not only to breathe when you have problems with the children but also when you have many problems in your head. So, learning to breathe well, to breathe deeply! Participant E*

*“The way of breathing at the moment when the children drive you crazy. This therapy of how to breathe to calm down.” Participant O*

### 3.5. Sustainability

The process evaluation found that the sustainability (and potential scale-up) of SA faces at least 3 important challenges: (1) guaranteeing the safety of the team, (2) procuring local support and commitment, and (3) ensuring funding to expand and maintain operations.

#### 3.5.1. Security

Tumaco is a highly complex territory where there is an amalgamation of social issues, including the presence of illegal armed groups, illegal drug trafficking, and forced recruitment and displacement. Confrontations between the existing legal and illegal armed groups generate invisible barriers at the territorial level. Therefore, entry into the neighborhoods posed the question if it was really possible to guarantee the safety of the implementation team. A facilitator mentioned:

*“Another challenge, that is also important, was the fear that I suppose many of us feel when entering certain places where violence is present. Where you didn’t know whether it was better to go in or not to go in. But love for the program, as well as our professionalism, helped us to keep moving forward”. Facilitator O*

Additionally, the interviewees mentioned that the necessity of developing strong partnerships and communication mechanisms with the communities where the program was going to be implemented to have their buy-in and support, prior to start any work. The facilitators highlighted the importance to engage key community stakeholders such as teachers, educators, government employees or other agents recognized and respected by the community. Facilitators underlined how constant interaction and communication with the community leaders helps to mitigate security risks.

*“We were going to the children’s kindergarten. ‘They’ [illegal armed-group members] knew that. We went in with our [program-labelled] vests; we brought our ID. They knew that we were there to help, contributing to the community, to the children. In other words, ‘They’ don’t interfere with aspects involving children. ‘They’ might get involved in other people’s affairs, but not the children’s: they also take care of the children. ‘They’ knew that we were not doing anything wrong, we weren’t spies of any kind or infiltrators; we were there to help the families, the children.” Facilitator H*

#### 3.5.2. Local support and commitment

The evidence gathered in the conversations with the team of facilitators revealed a few key challenges when scaling-out SA in other municipalities or territories. First, the facilitators mentioned establishing partnerships at the institutional level to ensure visibility and a better coordination of the program. A facilitator mentioned:

*“I think that the most important thing would be to foster connections with the main entity or institution in the municipality. Because we can’t arrive at a municipality with a program that no one knows about and simply go in and say: ‘okay, I’m going to work here and that’s that’. The idea is to foster those connections or partnerships once we arrive, similarly to when we arrive in the neighborhoods through the community action board. So, there we would connect with the Mayor and the [government] entities that exist in the territory. How can we discuss [with these partners about] the program? Obviously by […] asking for their perspectives about the municipality […]; what they think, whether they approve of the project taking place in the municipality.” Facilitator O*

#### 3.5.3. Funding

According to the technical team, the future donors of SA must take on a long-term commitment, of at least 3 years. That is the approximate time to reach the territories, adapt the curriculum to the context, and achieve the minimum quality level of training and implementation.

*“Well, the funding has always been… It’s a challenge for any program, right? And that determines many things, because once you are allied with the person that provides funding, well, after that we become responsible for what they want and how they want to measure progress and all of that, so having the flexibility to have funding that includes the level of support that we know works and reflective supervision is essential. These resources are necessary but worthwhile! We know that it has been a challenge to find partners that believe in this work and who want to develop high quality. And that it would be, a kind of commitment. We know that it takes time and that it would be a three-year commitment. At least three years, because normally the first year involves great efforts to connect with key people to start the training. It took pretty much that much time for them to truly internalize this, experience it, make it their own.” VR Technical Team*

## 4. Discussion

SA aims to fill a salient void in the portfolio of ECD services in Colombia, Latin America and other regions with ongoing armed conflicts or persistently high levels of community violence. Given the devastating effects of early exposure to violence, there is an urgency to expand the reach of evidence-based programs that can effectively promote resilience among families rearing children in contexts extreme adversity (Shonkoff, 2010; UNICEF, 2017). Building upon the successful experience of the CPP (Lieberman and Van Horn, 2011) and a thorough tailoring to the local culture, resources, and characteristics of victims of violence in Colombia (Molano et al., 2019), SA constitutes a scalable and sustainable effort to foster early-childhood development and protection in the context of community violence and forced displacement.

The process evaluation of the 2018–2019 phase of SA in Tumaco contributes to future efforts to expand ECD programs by advancing in the understanding of “threats to scalability” and enablers of “implementation success.” The evidence provided by this study suggests that the program’s curriculum is perceived as relevant and fit to the reality of participants, that caregivers have the intention to adopt the provided self-care and parenting tools, and that the overall level of fidelity is acceptable. Moreover, this study identifies two key implementation enablers that will be extrapolated to future scale-up and scale-out phases of SA, and that can be valuable to other ECD programs that aim at thriving in similar contexts.

The first implementation enabler was the intentional flexibility of the program for its adaptation to the context and culture. It is important to highlight that this adaptability is intentional, and follows a structured process that defines the contents in the curriculum that: (a) are core elements and “should never be adjusted” to the context; (b) “must be contextualized” every time the program lands into a new community, to promote appropriateness, adherence and effectiveness; and (c) “could be adapted,” if the implementation team perceives that the adjustment will improve the level of implementation success. This evaluation shows that a thorough and structured cultural adaptation is a strategic practice that promotes implementation success, and probably is also at the core of the program’s intervention success.

The second implementation enabler is the integration of a *task shifting* approach. Previous studies have demonstrated that task-shifting is particularly important and valuable in settings with few professionals available (Galvin and Byansi, 2020). Also, recent studies suggest that constraints on the locally available professionals is an important threat to scalability and partly explains differences in the observed impact of similar ECD programs across similar contexts, such as Jamaica, Colombia and Perú (Araujo et al., 2021). In the case of SA, having community agents as program facilitators was initially viewed as compulsory, given the unavailability of qualified professionals. However, the task-shifting approach has proven to be a fundamental enabler and a pilar for the program’s implementation success. The findings suggest that training community agents to deliver an evidence-based intervention is effective only when using innovative and appropriate teaching approaches such as experiential learning. Also, that the task-shifting strategy is only viable if there is a well-structured protocol that guarantees the continuous support by trained professionals to facilitators, all throughout the implementation of the program.

The results similarly suggest that task-shifting is not only valuable, but it is also a possibility in a low-resourced setting in Colombia, where there is a huge shortage of mental health professionals. By successfully training and supporting community health workers, the SA aims at building local capacity to develop a sustainable path to scale in places where: psychosocial support services are scarce (or inexistent) and, given the widespread of diverse expressions of violence, there is relatively large population of victimized families and children. Given that they already are part of the community, training lay health workers is particularly important because the intervention fosters an already existing trust and rapport with the participants, which is a highly important factor in conflict-affected areas where it is difficult to trust outsiders. Yet, it is important to highlight that the collected evidence suggests that the task-shifting model requires a robust process of recruitment, selection, and training of facilitators, and a structured mechanism that provides continuous support of program facilitators, such as the reflective supervision protocol.

Despite the encouraging findings, the study found that SA still has at least four areas of improvement. First, the safety of the work teams remains a concern in the implementation and scaling-up of the intervention. Previous studies have indicated that in contexts of violence, safety has consistently come up as a central topic in the ethics committees and has been subject to much supervision by universities and organizations (Sluka, 2020). While Tumaco could be cataloged as a difficult zone for implementation in terms of safety, the results of the implementation assessment suggest that a safety protocol that includes possible risks and solutions, must be developed before teams engage in field work.

Second, in terms of fidelity there is an important “learning curve” between the first time the program is implemented in a territory and the subsequent iterations. Evidence from the 2018–2019 implementation shows the dosage delivered to participants (measured with attendance rates and average total sessions attended) was much lower for the first cohort. This probably is the result of a combination of implementation threats, such as: lack of engagement and trust from the community and key allies (e.g., ECD Centers), inadequate training of the team, insufficiently deep adaptations to the context and culture, among other factors.

Third, the program needs to integrate differentiated strategies to prevent the low-dosage and higher drop-out rates of particularly vulnerable participants. For instance, the results from the statistical analysis show that participants that are less educated and have been direct victims of violence systematically have higher odds of dropping out of the program and not participating in enough sessions to benefit from the program. Improving the compliance of participants and attaining the minimum planned dosage for most (if not all) the participants that have an initial take-up is a direct way of improving the cost-effectiveness of the program.

The fourth area of improvement is related to the strengthening of the program’s monitoring system. In the evaluated phase of implementation (2018–2019), SA did not have any direct feedback channels with caregivers (i.e., the participants) to monitor program engagement and perception of the outcome. This is a key issue that should be addressed as a future improvement. An additional, and probably more challenging improvement opportunity for the monitoring system would be to develop an analytical tool to systematize the information exchanged in the reflective supervision sessions.

A recommended strategy to integrate the lessons learned in the first implementation efforts in Tumaco and prepare to expand the program to other communities, is to explicitly develop protocols around the four implementation stages proposed in the “EPIS conceptual model” (Aarons et al., 2011): (E)xploration stage, which includes all strategies for key stakeholder identification and engagement; (P)reparation stage, which includes a collaborative curriculum adaptation process and the procurement of strategic alliances and key input suppliers; (I)mplementation stage, that includes team selection and training processes, curriculum implementation, continuous team support and supervision and program monitoring; and (S)ustainment stage, which focuses on evidence production efforts (e.g., process evaluation), program adjustment and implementing a communication strategy to or extending the support of all key stakeholders.

Future implementation science studies could contribute by advancing designing and testing strategies to minimize the “learning curve” when deploying an evidence-based intervention in a new context. Future studies could also provide evidence on how to increase the cost-effectiveness of the program, which is a non-trivial determinant of scale-up efforts. Finally, subsequent research could integrate more the voices of participants to better understand their experience with the intervention and even have a *participatory action* approach by including caregivers in the study design.

## Data availability statement

The raw data supporting the conclusions of this article will be made available by the authors, without undue reservation.

## Ethics statement

All study procedures were reviewed and approved by the Institutional Review Board of Universidad de los Andes (record #1303, February 2021). The participants provided their written informed consent to participate in this study.

## Author contributions

AHR led the development of the field work and led the design and development of the manuscript. AHR conducted the analysis of the quantitative data. NCF and MP-L conducted the analysis of the qualitative data. AHR, NCF, and MP-L drafted the manuscript. All authors collectively conceived and conceptualized this original research article, contributed to the discussion, reviewed the manuscript, and provided critical feedback.

## Funding

We acknowledge generous financial support the Saving Brains partnership (Grant Agreement ID SB-POC-1809-19091), United Way Colombia, Fundación Exito, Fundacion FEMSA, Genesis Foundation, Fundación Coca-Cola, Primero lo Primero, and the Facultad de Typo: Economía at Universidad de los Andes.

## Conflict of interest

The authors declare that the research was conducted in the absence of any commercial or financial relationships that could be construed as a potential conflict of interest.

## Publisher’s note

All claims expressed in this article are solely those of the authors and do not necessarily represent those of their affiliated organizations, or those of the publisher, the editors and the reviewers. Any product that may be evaluated in this article, or claim that may be made by its manufacturer, is not guaranteed or endorsed by the publisher.

## Figures and Tables

**Table:** 

Session	Main objective of core activity
Module 1	Promoting maternal health
Session 1	Recognizing the importance of non-judgmental and accepting relationships, as the base of constructive processes.
Session 2	Recognizing personal strengths and skills to promote self-esteem and group integration.
Session 3	Increasing reflexive capacity to identify the roles assumed in life and better understand how they have impacted family dynamics and parenting.
Session 4	Increasing awareness of how parenting choices and practices impact who their children will be in the future.
Session 5	Reflecting on the possibility of adopting an alternative childrearing pattern, different from the way they were raised.
Session 6	Recognizing the impact of experiences of violence, and reflecting on the present needs and future actions.
Session 7	Recognizing the each person’s journey so far and the impact on the upbringing of their sons and daughters.
Module 2	Promoting early childhood development
Session 8	Recognizing the responsibility and capacity to ensure a nurturing environment, protection and safety to their children.
Session 9	Identifying assertive ways of relating with their children, given the stages of child development.
Session 10	Understanding basic principles of child development and how children communicate their needs through behavior.
Session 11	Learning strategies to respond appropriately to children’s needs.
Session 12	Increasing skills and confidence when talking to children about adverse life experiences (potentially traumatic).
Module 3	Strengthening social support networks
Session 13	Increasing confidence to engage other adults to create a childrearing team and a support network.
Session 14	Increasing reflective capacity on the impact of changes and transitions on their own life and on their childrens’ lives.
Session 15	Increasing reflective capacity on closure and farewells, for themselves and their children.

## Appendix

APPENDIX A Main objectives of the modules and sessions in the Semillas de Apego curriculum.

## References

[ref1] Aarons G. A. Hurlburt M. Horwitz S. M. (2011). Advancing a conceptual model of evidence-based practice implementation in public service sectors. Admin. Pol. Ment. Health 38, 4–23. doi: 10.1007/s10488-010-0327-7, PMID: 21197565 PMC3025110

[ref2] Aboud F. E. Yousafzai A. K. Nores M. (2018). State of the science on implementation research in early child development and future directions. Ann. N. Y. Acad. Sci. 1419, 264–271. doi: 10.1111/nyas.13722, PMID: 29791728

[ref3] Allison P. D. (1984). “Proportional hazards and partial likelihood” in Event history analysis (Thousand Oaks, CA: SAGE Publications, Inc.), 34–42. doi: 10.4135/9781412984195.n4

[ref4] Allison PD. (2014). Event history and survival analysis: Regression for longitudinal event data. Available at: https://books.google.com.co/books?id=_DJ1AwAAQBAJ

[ref5] Al-Ubaydli O. Lee M. S. List J. A. Suskind D. (2021). “The science of using science: a framework for understanding the threats to scaling evidence-based policies” in The scale-up effect in early childhood & public policy.: Why interventions lose impact at scale and what we can do about it. eds. List J. Suskind D. Suplee L. (New York, NY: Routledge)

[ref6] Araujo M. C. Rubio-Codina M. Schady N. (2021). “70 to 700 to 70,000: lessons from the Jamaica experiment” in The scale-up effect in early childhood & public policy: Why interventions lose impact at scale and what we can do about it. eds. List J. Suskind D. Suplee L. (New York, NY: Routledge) doi: 10.4324/9780367822972-15

[ref7] Barron C. C. Dayton C. J. Goletz J. L. (2022). From the voices of supervisees: what is reflective supervision and how does it support their work? (part I). Infant Ment. Health J. 43, 207–225. doi: 10.1002/imhj.21972, PMID: 35165913

[ref8] Black M. M. Walker S. P. Fernald L. C. H. Andersen C. T. DiGirolamo A. M. Lu C.. (2017). Early childhood development coming of age: science through the life course. Lancet 389, 77–90. doi: 10.1016/S0140-6736(16)31389-7 27717614 PMC5884058

[ref9] Blair C. (2010). Stress and the development of self-regulation in context. Child Dev. Perspect. 4, 181–188. doi: 10.1111/j.1750-8606.2010.00145.x, PMID: 21779305 PMC3138186

[ref10] Borghans L. Duckworth A. Heckman J. Ter Weel B. (2008). The economics and psychology of personality traits. J. Hum. Resour. 43, 972–1059. doi: 10.1353/jhr.2008.0017

[ref11] Bowles S. Gintis H. Osborne M. (2001). The determinants of earnings: a behavioral approach. J. Econ. Lit. 39, 1137–1176. doi: 10.1257/jel.39.4.1137

[ref14] Buccini G. Venancio S. I. Pérez-Escamilla R. (2021). Scaling up of Brazil's Criança Feliz early childhood development program: an implementation science analysis. Ann. N. Y. Acad. Sci. 1497, 57–73. doi: 10.1111/nyas.14589, PMID: 33738809 PMC8349773

[ref15] Cawley J. Heckman J. Vytlacil E. (2001). Three observations on wages and measured cognitive ability. Labour Econ. 8, 419–442. doi: 10.1016/S0927-5371(01)00039-2

[ref05] Cicchetti D. Toth S. L. Rogosch F. A. (1999). The efficacy of toddler-parent psychotherapy to increase attachment security in offspring of depressed mothers. Attach. Hum. Dev. 1, 34–66. doi: 10.1080/14616739900134021 11707882

[ref16] Dusenbury L. Brannigan R. Falco M. Hansen W. B. (2003). A review of research on fidelity of implementation: implications for drug abuse prevention in school settings. Health Educ. Res. 18, 237–256. doi: 10.1093/her/18.2.237, PMID: 12729182

[ref17] Englander M. (2012). The interview: data collection in descriptive phenomenological human scientific research. J. Phenomenol. Psychol. 43, 13–35. doi: 10.1163/156916212X632943

[ref18] Erdemir E. (2022a). Home-based early education for refugee and local children via mothers: a model of contextually sensitive early intervention. J. Child Fam. Stud. 31, 1121–1144. doi: 10.1007/s10826-021-02197-7

[ref19] Erdemir E. (2022b). Uncovering community cultural wealth through an early intervention program: Syrian refugee children speaking. Early Childhood Educ. J. 50, 259–278. doi: 10.1007/s10643-020-01140-7

[ref20] Evans G. Schamberg M. (2009). Childhood poverty, chronic stress, and adult working memory. Proc. Natl. Acad. Sci. 106, 6545–6549. doi: 10.1073/pnas.0811910106, PMID: 19332779 PMC2662958

[ref22] Felitti V. (2002). The relation between childhood experiences and adult health: turning gold into lead. Perm. J. 6, 44–47. 30313011 10.7812/tpp/02.994 PMC6220625

[ref23] Galvin M. Byansi W. (2020). A systematic review of task shifting for mental health in sub-Saharan Africa. Int. J. Ment. Health 49, 336–360. doi: 10.1080/00207411.2020.1798720

[ref24] Gonçalves T. R. Duku E. Janus M. (2019). Developmental health in the context of an early childhood program in Brazil: the "Primeira Infância Melhor" experience. Cad. Saude Publica. 35:e00224317. doi: 10.1590/0102-, PMID: 30864615

[ref04] Guild D. J. Toth S. L. Handley E. D. Rogosch F. A. Cicchetti D. (2017). Attachment security mediates the longitudinal association between child-parent psychotherapy and peer relations for toddlers of depressed mothers. Dev. Psychopathol. 29, 587–600. doi: 10.1017/S0954579417000207 28401848 PMC5586600

[ref25] Gupta S. Supplee L. H. Suskind D. List J. A. (2021). “Failed to scale: embracing the challenge of scaling in early childhood” in The scale-up effect in early childhood & public policy. Why interventions lose impact at scale and what we can do about it. eds. List J. Suskind D. Suplee L. (New York, NY: Routledge) doi: 10.4324/9780367822972-1

[ref26] Harker Roa A Moya A Camperos LM. (2017). Semillas de Apego: Un Programa para la Protección de la Primera Infancia Víctima del Desplazamiento Forzado y la Violencia en Colombia. Informe de Final Preparado para el Banco Interamericano de Desarrollo - BID. Dirección de Políticas Públicas para la Prevención y Reducción de la Criminalidad en Áreas Urbanas de América Latina y el Caribe. Unpublished Document.

[ref27] Heckman J. (2006). Skill formation and the economics of investing in disadvantaged children. Science 312, 1900–1902. doi: 10.1126/science.1128898, PMID: 16809525

[ref28] Hogue A. Liddle H. A. Rowe C. (1996). Treatment adherence process research in family therapy: a rationale and some practical guidelines. Psychotherapy 33, 332–345. doi: 10.1037/0033-3204.33.2.332

[ref29] Ibáñez A. M. Moya A. (2010). Vulnerability of victims of civil conflicts: empirical evidence for the displaced population in Colombia. World Dev. 38, 647–663. doi: 10.1016/j.worlddev.2009.11.015

[ref30] Ippen C. G. Harris W. H. Van Horn P. Lieberman A. F. (2011). Traumatic and stressful events in early childhood: can treatment help those at highest risk? Child Abuse Negl. 35, 504–513. doi: 10.1016/j.chiabu.2011.03.009, PMID: 21816474 PMC3159839

[ref32] Lewis C. C. Fischer S. Weiner B. J. Stanick C. Kim M. Martinez R. G. (2015a). Outcomes for implementation science: an enhanced systematic review of instruments using evidence-based rating criteria. Implement. Sci. 10:155. doi: 10.1186/s13012-015-0342-x, PMID: 26537706 PMC4634818

[ref33] Lewis C. C. Stanick C. F. Martinez R. G. Weiner B. J. Kim M. Barwick M.. (2015b). The society for implementation research collaboration instrument review project: a methodology to promote rigorous evaluation. Implement. Sci. 2015 10:2. doi: 10.1186/s13012-014-0193-x 25567126 PMC4308900

[ref34] Lieberman A. F. Ippen C. G. Van Horn P. (2006). Child-parent psychotherapy: 6-month follow-up of a randomized controlled trial. J. Am. Acad. Child Adolesc. Psychiatry 45, 913–918. doi: 10.1097/01.chi.0000222784.03735.92, PMID: 16865033

[ref35] Lieberman AF Van Horn P. (2011). Psychotherapy with infants and young children: Repairing the effects of stress and trauma on early attachment, New York: Guilford Press.

[ref37] Meneses Copete Y. A. (2022). Alabaos Y currulaos: resignificación de prácticas identitarias Y culturales afropacíficas en El Marco Del Conflicto Armado. Investig. Desarro. 30, 374–407. doi: 10.14482/INDES.30.1.305.8

[ref38] Molano A. Harker Roa A. Cristancho J. C. (2018). Effects of indirect exposure to homicide events on Children’s mental health: evidence from urban settings in Colombia. J. Youth Adolesc. 47, 2060–2072. doi: 10.1007/s10964-018-0876-8, PMID: 29948860

[ref39] Molano A Harker Roa A., Moya A Camperos LM Gamboa LA Niño B Y Reyes V. (2019). Evaluación de la calidad en la implementación del programa Semillas de Apego: Análisis documental de la fidelidad al diseño curricular. Universidad de los Andes. Unpublished document.

[ref40] Moya A Lieberman AF Harker Roa A Niño B Sanchez J Torres MJ. (2022). Maternal health and early-childhood development in conflict-affected settings: Experimental evidence from Colombia. Working Paper. Unpublished document.

[ref06] Nathaniel A. (2021). From the editor’s desk: Classic Grouded Theory: What it is and what it is not. Grounded Theory Review 20, 1–7.

[ref41] Noble K. G. (2021). “Early childhood: the opportunity to untap human potential” in The scale-up effect in early childhood & public policy.: Why interventions lose impact at scale and what we can do about it. eds. List J. Suskind D. Suplee L. (Routledge)

[ref42] Orkin A. M. Rao S. Venugopal J. Kithulegoda N. Wegier P. Ritchie S. D.. (2021). Conceptual framework for task shifting and task sharing: an international Delphi study. Hum. Resour. Health 19:61. doi: 10.1186/s12960-021-00605-z, PMID: 33941191 PMC8091141

[ref43] Ortega JA. (2018). Metodología del ranking (2017) de las 50 ciudades más violentas del mundo. Seguridad Justicia y Paz – Consejo Ciudadano para la Seguridad Pública y Justicia Penal A.C. Available at: https://www.oas.org/ext/es/seguridad/red-prevencion-crimen/Recursos/Biblioteca-Digital/ranking-2017-de-las-50-ciudades-mas-violentas-del-mundo

[ref44] Proctor E. K. Landsverk J. Aarons G. Chambers D. Glisson C. Mittman B. (2009). Implementation research in mental health services: an emerging science with conceptual, methodological, and training challenges. Adm. Policy Ment. Health Ment. Health Serv. Res. 36, 24–34. doi: 10.1007/s10488-008-0197-4, PMID: 19104929 PMC3808121

[ref45] Proctor E. Silmere H. Raghavan R. Hovmand P. Aarons G. Bunger A.. (2011). Outcomes for implementation research: conceptual distinctions, measurement challenges, and research agenda. Adm. Policy Ment. Health Ment. Health Serv. Res. 38, 65–76. doi: 10.1007/s10488-010-0319-7, PMID: 20957426 PMC3068522

[ref46] R4V/Inter-Agency Coordination Platform for Migrants and Refugees from Venezuela. (2022). Regional refugee and migrant response plan (RRMRP). January 2023–December 2024. Available at: https://www.r4v.info/en/rmrp2023-2024

[ref47] Reyes Vilma Lieberman Alicia, Building bridges: Child-parent psychotherapy group model (2010).

[ref48] RUV – Registro Único de Víctimas. (2022). Red Nacional de Información, RUV. Unidad Para la Atención y Reparación Integral a las Víctimas. Available at: https://www.unidadvictimas.gov.co/es/registro-unico-de-victimas-ruv/37394

[ref49] Shonkoff J. P. (2010). Building a new biodevelopmental framework to guide the future of early childhood policy. Child Dev. 81, 357–367. doi: 10.1111/j.1467-8624.2009.01399.x, PMID: 20331672

[ref50] Shonkoff J Phillips D. (2000). From neurons to neighborhoods: The science of early childhood development. Washington, DC: National Academy Press. 25077268

[ref08] Sluka J. A. (2020). Too dangerous for fieldwork? The challenge of institutional risk-management in primary research on conflict, violence and ‘Terrorism’. Contemp. Soc. Sci. 15, 241–257. doi: 10.1080/21582041.2018.1498534

[ref51] Smith J. A. Baker-Henningham H. Brentani A. Mugweni R. Walker S. P. (2018). Implementation of reach up early childhood parenting program: acceptability, appropriateness, and feasibility in Brazil and Zimbabwe. Ann. N. Y. Acad. Sci. 1419, 120–140. doi: 10.1111/nyas.13678, PMID: 29791729

[ref52] Strauss AL. (1990). Qualitative analysis for social scientists. New York: Cambridge University Press.

[ref53] Strauss AL Corbin J. (2002). Bases de la investigación cualitativa: técnicas y procedimientos para desarrollar la teoría fundada (1st). Medellín: Editorial Universidad de Antioquia.

[ref01] Strømme A. Fylkesnes G. K. Denselow J. Mangan R. Podieh P. Kamøy K. (2022). “Stop the War on Children: The forgotten ones”. Save the Children International.

[ref54] Susman-Stillman A. Lim S. Meuwissen A. Watson C. (2020). Reflective supervision/consultation and early childhood professionals’ well-being: a qualitative analysis of supervisors’ perspectives. Early Educ. Dev. 31, 1151–1168. doi: 10.1080/10409289.2020.1793654

[ref02] Toth S. L. Maughan A. Manly J. T. Spagnola M. Cicchetti D. (2002). The relative efficacy of two interventions in altering maltreated preschool children’s representational models: implications for attachment theory. Dev. Psychopathol. 14, 877–908. doi: 10.1017/s095457940200411x 12549708

[ref03] Toth S. L Sturge-Apple M. L. Rogosch F. A. Cicchetti D. (2015). Mechanisms of change: Testing how preventative interventions impact psychological and physiological stress functioning in mothers in neglectful families. Dev. Psychopathol. 27, 1661–1674. doi: 10.1017/S0954579415001017 26535951 PMC4633703

[ref55] UNHCR. (2022). Global trends forced displacement in 2021.

[ref56] UNICEF. (2017). Violence in early childhood. Regional framework for UNICEF in Latin America and the Caribbean. ECD and child protection sections – UNICEF LACRO.

[ref57] UNODC (2019). Global study on homicide. Available at: https://www.unodc.org/unodc/en/data-and-analysis/global-study-on-homicide.html

[ref58] Walker S. Wachs T. Gardner J. M. Lozoff B. Wasserman G. Pollitt E.. (2007). Child development: risk factors for adverse outcomes in developing countries. Lancet 369, 145–157. doi: 10.1016/S0140-6736(07)60076-2 17223478

[ref59] WHO. (2008). Task shifting: Rational redistribution of tasks among health workforce teams – global recommendations and guidelines. World Health Organization – PEPFAR – UNAIDS. NLM classification: WC 503.6

[ref60] WHO and ExpandNet. (2010). Nine steps for developing a scaling-up strategy. World Health Organization. Available at: https://apps.who.int/iris/handle/10665/44432

[ref61] Wilson K. Weatherston D. J. Hill S. (2021). “Introduction to reflective supervision: through the lens of culture, diversity, equity, and inclusion” in Therapeutic cultural routines to build family relationships. eds. Lewis M. L. Weatherston D. J. (Cham: Springer).

